# Preparation, characterization, and *in vitro*/*vivo* evaluation of dexamethasone/poly(ε-caprolactone)-based electrode coatings for cochlear implants

**DOI:** 10.1080/10717544.2021.1960927

**Published:** 2021-08-04

**Authors:** Yanjing Luo, Anning Chen, Muqing Xu, Dongxiu Chen, Jie Tang, Dong Ma, Hongzheng Zhang

**Affiliations:** a Department of Otolaryngology Head & Neck Surgery, Zhujiang Hospital, Southern Medical University, Guangzhou, China; b Hearing Research Center, Southern Medical University, Guangzhou, China; c Department of Physiology, School of Basic Medical Sciences, Southern Medical University, Guangzhou, China; d Key Laboratory of Mental Health of the Ministry of Education, Southern Medical University, Guangzhou, China; e Key Laboratory of Biomaterials of Guangdong Higher Education Institutes, Department of Biomedical Engineering, Jinan University, Guangzhou, China

**Keywords:** Poly(ε-caprolactone), cochlear implantation, electrode coating, dexamethasone

## Abstract

With dexamethasone as the model drug and polycaprolactone (PCL) as the carrier material, a drug delivery coating for cochlear electrodes was prepared, to control cochlear fibrosis caused by cochlear implantation. A dexamethasone/poly (ε-caprolactone)-based electrode coating was prepared using the impregnation coating method. Preparation parameters were optimized, yielding 1 impregnation instance, impregnation time of 10 s, and PCL concentration of 10%. The coating was characterized *in vitro* using scanning electron microscopy, a universal machine, high-performance liquid chromatography, and CCK-8. The surface was porous and uniformly thick (average thickness, 48.67 µm)—with good flexibility, long-term slow drug release, and optimal drug concentration—and was biologically safe. The experimental results show that PCL is an ideal controlled-release material for dexamethasone as a drug carrier coating for cochlear implants.

## 1. Introduction

Cochlear implantation (CI) is the main treatment method for patients suffering from severe sensorineural hearing loss. Cochlear implants can transform acoustic signals into electrical signals to stimulate auditory neurons via an array of electrodes implanted into the cochlea (Richardson et al., 2009). Furthermore, hybrid cochlear implants that combine acoustic and electric stimulation approaches can provide novel treatment pathways for patients with residual hearing at low frequencies (Kikkawa et al., 2014). Thus, it is imperative to preserve presurgical residual hearing in these patients, because it is critical for hybrid cochlear implants systems (Kiefer et al., 2004; Turner et al., 2008).

However, it has been reported that patients are likely to lose residual hearing at low frequencies within a few months after cochlear implantation (Jia et al., 2016). Two factors are considered to be important contributors to this residual hearing loss: (1) inflammatory responses caused by mechanical trauma during the implantation process, which can lead to cochlear fibrosis, and (2) fibrosis and new bone formation in the cochlea, which can increase electrode impedance, thus compromising the survival of auditory hair cells (HCs) and neurons (Fayad et al., 2009; Bas et al., 2012; Jia et al., 2013; Ceschi et al., 2014; Bas et al., 2017; Eftekhari et al., 2020; Kather et al., 2021). A possibility of damage to the stria vascularis (SV) has been previously reported, revealing a significant correlation between the residual hearing loss and blood vessel density in the SV (Tanaka et al., 2014). Therefore, to preserve residual hearing and avoid the obstruction of re-implantation, it is necessary to suppress the inflammatory response and prevent cochlear fibrosis after surgery.

Glucocorticoids play an important role in the treatment of inflammations. Previous *in vivo* studies revealed that glucocorticoids can effectively reduce cochlear damage and hearing loss, caused by trauma or ototoxic drugs (Takemura et al., 2004; Zou et al., 2005; Tabuchi et al., 2006). The most commonly used corticosteroids include dexamethasone (DXM), prednisone, and methylprednisolone. These drugs can activate anti-inflammatory and anti-apoptotic signals in the ear (Chandrasekhar, 2001; Himeno et al., 2002; Daldal et al., 2007). They, especially DXM, are widely used for treating various conditions after cochlear implantation. Previous animal model studies have shown that DXM can reduce hearing loss and cochlear damage that is caused by cochlear implantation (Eshraghi et al., 2007; Vivero et al., 2008). Furthermore, previous studies revealed that corticosteroids directly applied to the scala tympani during cochlear implantation can prevent fibrosis (De Ceulaer et al., 2003; Paasche et al., 2009; Enticott et al., 2011). Moreover, in the guinea pig model, DXM inhibited inflammation and fibrous tissue around the implant post-surgery (Lee et al., 2013; Astolfi et al., 2016). However, there is a blood-labyrinth barrier in the inner ear, which is similar to the blood-brain barrier, and this barrier can prevent intravenously administered drugs from reaching the inner ear effectively. Thus, when corticosteroids were administered systemically *via* the intravenous route after surgery, the treatment was less efficient (Jia et al., 2011). In addition, studies have shown that corticosteroids directly applied to the inner ear during cochlear implantation can prevent fibrosis (De Ceulaer et al., 2003; Paasche et al., 2009; Enticott et al., 2011). However, for all administered drugs, the therapeutic effects of corticoids were short-lived, owing to their short half-life time *in vivo* (Enticott et al., 2011).

To improve the impact of corticoids, a system for drug release that delivered DXM directly to the scala tympani through the round window membrane has been used in animal models; this approach has been shown to reduce a residual hearing loss (Eshraghi et al., 2007; Vivero et al., 2008). However, owing to the limited permeability of the cochlear membrane and the special structure of the cochlea, the drug concentration reaching the cochlea using this delivery system is uneven and not controllable and (Astolfi et al., 2016). In addition, some researchers have reported that various drug delivery devices can release drugs directly into the inner ear. Examples include the drug-loaded elastomeric silicone electrode and the DXM-eluting electrode (Farhadi et al., 2013; Astolfi et al., 2014; Astolfi et al., 2016). However, as the drug is released, tiny pores gradually appear on the surface of the delivering electrode, which could destroy the integrity of the electrode, eventually impairing the electrode’s conductivity and affecting the cochlear implant’s efficiency (Stathopoulos et al., 2014). Research also showed that the amount of DXM released from the DXM-eluting electrode was insufficient for the treatment of hearing loss induced by the insertion trauma (O'Leary et al., 2013). Our previous results suggest that drug-based electrode coatings are effective for administering drugs to the inner ear. This method of administration, combined with cochlear implant electrodes, could reduce the aggressiveness of administration. However, we found that it is difficult to control the coating’s thickness, which determines the actual drug loading of the coating; as a result, the drug’s dose released into the inner ear is difficult to control (Xu et al., 2018). Thus, the objective of the present study was to design a drug-loading electrode coating for DXM delivery after cochlear implantation. The coating should have the proper thickness to carry enough drugs without affecting the electrode’s performance, and the drug should be released in a sustained manner for an effective long-term treatment.

Poly-ε-caprolactone (PCL) is a linear aliphatic polyester prepared by ring-opening polymerization of ε-caprolactone (ε-CL), which has garnered significant attention as an ideal material for drug delivery systems (DDSs) and tissue engineering applications (Mondrinos et al., 2006; Seregin and Coffer, 2006). Studies have shown that PCL is an excellent drug delivery carrier, owing to its good drug-loading capability (Xu et al., 2018). Owing to its high biocompatibility, PCL was approved by the Food and Drug Administration (FDA) in 1954 and has been used for manufacturing implantable medical devices (Rohner et al., 2002; Teoh et al., 2004). Furthermore, just grounded on the special chemical structure character, PCL exhibits excellent processability, preferential mechanical properties, and a relatively slow rate of degradation. New nanomaterials are a promising development direction in tissue engineering. One study found that PCL is often combined with other more brittle materials, for enhancing the stress cracking resistance, owing to its good toughness (Nair and Laurencin, 2007). Animal model studies suggest that PCL decomposes into smaller molecular fragments after 30 months, does not accumulate in the body, and is eventually completely excreted (Sun et al., 2006).

In the present study, 5, 10, and 20% DXM were incorporated into PCL mixtures which were mixed in different ratios. The objective of this study was to develop a drug-loaded electrode coating, which should be biocompatible, exhibit a certain biodegradation rate, good drug-loading capability, and controllable thickness. Specific properties used for evaluating the performance of the coating were surface/microscopic morphology, mechanical properties, and *in vitro* and *in vivo* biological safety. Furthermore, the controllability of PCL coating and the capability to release slowly and support long-term treatment effects were also investigated using different parameters of the dip-coating method, such as the solution concentration, immersion time, number of repeats, and the amount of the drug-loaded.

## 2. Materials and methods

### 2.1. Materials

The high molecular weight Poly-ε-caprolactone (PCL) (pellets, Mw = 80 kDa) and high molecular weight PCL (pellets, Mw = 60 kDa) were obtained from Dalian Meilun Biological Technology Co., Ltd., Dalian, China. High molecular weight PCL (pellets, Mw = 36 kDa) was provided by Aikeda Chemical Reagent Company in Chengdu, China. Low molecular weight PCL (powders, Mw = 2 kDa) was supplied by HEOWNS (Tianjin, China). A number after ‘PCL’ refers to its molecular weight. Dexamethasone (DXM, purity ≥ 98%) was from Meryer Inc. (Shanghai, China). Cytotoxicity was evaluated with a Cell Counting kit-8 (Dojindo, Japan). The regents used in high-performance liquid chromatography (HPLC, Agilent 1260, USA) were of chromatographic grade, and the other chemical reagents used were of analytical grade.

### 2.2. Preparation of drug-loaded blend PCL coating

**Figure 1.**
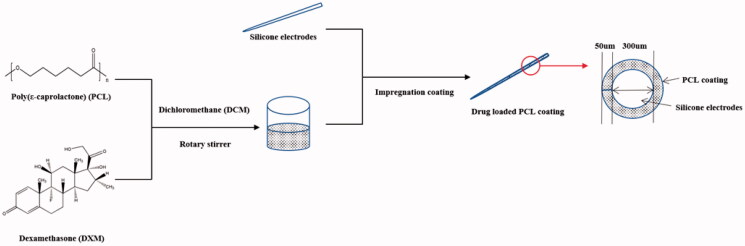
Schematic of the preparation procedures of DXM/PCL-based electrode coatings.

A kind of silicone rod was used to simulate the electrodes. The cylindrical rod had a length of 8 cm and three kinds of diameters = 1, 4, and 8 mm, respectively (Figure 1). Different molecular weight PCL binary mixtures were prepared as follows. High molecular weight PCL and low molecular weight PCL at 90:10, 80:20, 60:40, 50:50 wt% were weighed in the sample bottle. DXM was added into PCL mixtures with four different concentrations. PCL mixtures: DXM mass ratio was 95:5, 90:10, and 80:20. Dichloromethane (DCM) was used for preparing all 10% (w/w) DXM/PCL coating solutions since it enabled the dissolution of hydrophobic polymer and drugs in this study. DXM/PCL coating solution was prepared by dissolving PCL pellets/powder and DXM mixtures in DCM using magnetic stirring at room temperature and a stirring speed of 500 rpm. When all the DXM powder and PCL pellets/powder had been dissolved in mixed solution, indicating that the dissolution process is finished. Silicone rods were totally immersed in the DXM/PCL coating solution. After certain immersion times, each silicone rod was removed and suspended to a holder in the fume hood at room temperature for 24 h to facilitate solvent evaporation.

Preliminary researches by the authors indicated that the maximum dissolution saturation for PCL in DCM was ∼16 wt%. And at 14 wt% the coating solution was more viscous and harder to prepared uniform coating. Therefore, 12 wt% was selected as the upper limit for PCL concentration in this study. To research the optimal PCL concentration, at equal intervals other concentration levels below the upper limit were selected. In addition, preliminary studies by the authors indicated that a prolonged immersion time would cause an uneven coating thickness. So, to study the best immersion period for electrodes to be coated with PCL, a relatively short range of immersion times was selected. And by repeating the same steps as describes for the first layer, layers of PCL were applied onto each electrode.

In summary, during the coating preparing procedure the following preparation parameters and levels were investigated by evaluation of the coating thickness and uniformity: Molecular weight of PCL (2, 36, 60, and 80 kDa), Ratio of different molecular weights of PCL (10:90, 20:80, 40:60, and 50:50), PCL concentration (2, 4, 6, 8, 10, and 12 wt%), Immersion time (1, 10, and 30 s) and Number of dip coating (1, 3, and 5).

### 2.3. Scanning electron microscopy observation

Each DXM/PCL coating was cut into 5 × 5 mm for ultra-structure analysis, fixed onto the metallic studs with double-sided conductive tape. The surface of the coatings was sputter-coated with gold. And subsequently, the morphological microstructure of coating, such as the surface of DXM/PCL coating, was studied by a high-resolution scanning electron microscope (SEM, Zeiss ULTRA 55).

### 2.4. Characterization of coating’s mechanical properties

The silicone rod which had a diameter of 4 mm and was coated with different DXM/PCL coating was cut into 75 mm for mechanical property analysis. The tensile curve of the DXM/PCL coating was measured by an electrical universal material testing machine (ElectroForce, BOSE, USA). The samples were fixed between two clamps with a 6 cm gap. And the tensile rate was 3 mm/min. The tensile test date was used to linear fitting and educed the stress-strain curve. And the slope of the stress-strain was obtained as the young’s modulus of the tensile sample.

### 2.5. Dexamethasone release profile

The silicone rod which had a diameter of 8 mm and was coated with different DXM/PCL coating was cut into 3.5 cm for release profile analysis. Each sample was placed in individual non-sterile polypropylene 5 mL vials containing 3.8 mL artificial perilymph (APL). Each container was placed in an incubator shaker that was maintained at 37 °C and 100 rpm for 300 days. At predetermined time points, the pre-determined volume of the solution was withdrawn and replenished with fresh solution. DXM release tests were performed in triplicate. The extracted samples were filtered through 0.2 μm nylon filters and analyzed using HPLC (Agilent 1260, USA). The mobile phase consisted of a mixture of water: acetonitrile 73:27 (v/v), and the flow rate was set at 1 mL/min. UV detection was set at 275 nm, the column temperature was maintained at 25 °C. The injection volume was 20 μL, and the analytical run time for each sample was 16 min. Initial standard stock solutions of DXM were diluted with an APL to produce six standard solutions in the range of 0.01–1 mg/mL. A linear relationship between the DXM concentration and peak area was obtained with a correlation coefficient of 0.9987. The area under the peak was used for calculating the DXM concentration in the samples.

### 2.6. *In vitro* biocompatibility test

The safety and biocompatibility of the coatings were evaluated on mouse fibroblast cells (L929). Fibroblasts were cultivated in RPMI 1640 cell culture medium supplemented with 10% fetal bovine serum (FBS), 1% penicillin, and streptomycin. Cells were maintained in an incubator at 37 °C and CO2. In the next experiment setting, safety and biocompatibility were measured by the indirect method. By the indirect method, 12 different types of coatings were incubating in PBS for 24 h to prepare the leaching liquor. In this case, taking PBS as a negative control, and cisplatin solution (2 mg/mL) as a positive control, the cell counting kit-8 (CCK-8, Dojindo, Japan) was used to evaluate the cell viability of L929 in the leaching liquor. The cells were grown in 96 well-plates with 5000 cells per well and grown in 100 μl culture medium for 24 h, next incubated with 10 μl of PBS, cisplatin solution, or different leaching liquors for 24 h, respectively. Then to perform the CCK-8 assay, each well was replaced with 100 μL serum-free RPMI 1640 and incubated with 10 μL of CCK-8 agent. Then the plates were incubated in the 5% CO2 incubator for 2 h at 37 °C. The absorbance value at 450 nm was determined by using the microplate reader (BioTek ELx808, USA).

### 2.7. *In vivo* biocompatibility test

#### 2.7.1. Subcutaneous implant

Eighty healthy rats (6 weeks old, 200–250 g each) were used as experimental animals. The protocol was approved by the Laboratory Animal Ethical Committee of Zhujiang Hospital of Southern Medical University, and the experiment was conducted according to the committee guidelines. All the coatings samples (0.5*1 cm) were sterilized by soaking in 75% alcohol for 2 h, then by ultraviolet radiation. The rats were anesthetized with pentobarbital sodium, and their backs were shaved. Then they were sterilized with 75% alcohol and iodine scrubs. Two paravertebral incisions (about 2 cm each) per rat were made ∼1 cm lateral to the vertebral column to expose the dorsal subcutis. Then subcutaneous pocks were created by blunt dissection. Each individual pocket held one coating sample. And the incisions were closed with surgical sutures. All surgeries were carried out in an aseptic field by using the aseptic technique. A total of samples 112 samples (*n* = 4 for each experimental group) for each of the coatings were implanted.

#### 2.7.2. Histology

The biocompatibility and the effect on the tissue of the coating samples were evaluated till 3 weeks of implantation. After post-surgery 1 week and 3 weeks, one animal from each group was euthanized, and the implants were harvested with surrounding tissue for examination. The harvested implants and tissue were fixed in 4% paraformaldehyde solution for 24 h. Then they were dehydrated in a graded series of ethanol and embedded in paraffin. Then the explants were sectioned (4 μm) in a transversal direction from arbitrary regions perpendicular to the long axis of the tissue capsule by using a standard microtome. The paraffin sections were stained with hematoxylin and eosin (H&E) for morphological evaluation. Then the sections were observed under a light microscope.

## 3. Results and discussion

The present study reports the preparation and characterization of a coating that can release DXM in a controlled manner. The form of the electrode coating allows to release DXM in a prolonged and tunable manner *in situ*, which enables a more efficient therapy in terms of the administered drug dose. Importantly, the DXM release can be assured along the scala tympani. The proposed preparation method allows to fine-tune the coating, to meet the specific mechanical and drug dosing requirements of the therapeutic approach.

### 3.1. Morphology of coatings

**Figure 2.**
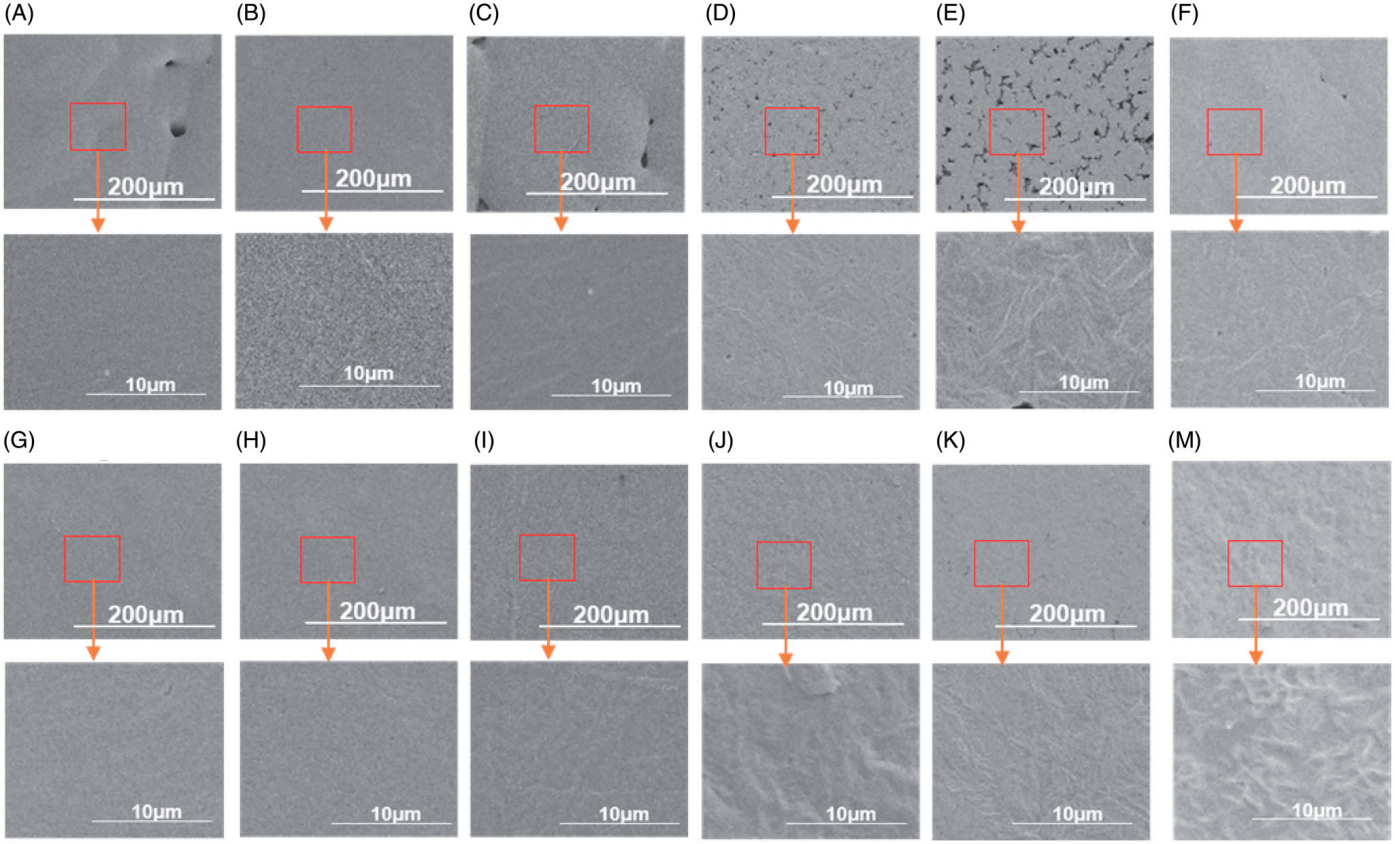
The surface SEM images of PCL coatings prepared under various treatment conditions. (A) PCL_Mw = 2 kDa:36 kDa_ coating without drug and loaded with 10% PCL_Mw = 2 kDa_, (B) PCL_Mw = 2 kDa:36 kDa_ coating without drug and loaded with 20% PCL_Mw = 2 kDa_, (C) PCL_Mw = 2 kDa:36 kDa_ coating without drug and loaded with 40% PCL_Mw = 2 kDa_, (D) PCL_Mw = 2 kDa:36 kDa_ coating with 10% DXM and loaded with 20% PCL_Mw = 2 kDa_, (E) PCL_Mw = 2 kDa:36 kDa_ coating with 10% DXM and loaded with 40% PCL_Mw = 2 kDa_, (F) PCL_Mw = 2 kDa:60 kDa_ coating without drug and loaded with 40% PCL_Mw = 2 kDa_, (G) PCL_Mw = 2 kDa:80 kDa_ coating without drug and loaded with 10% PCL_Mw = 2 kDa_, (H) PCL_Mw = 2 kDa:80 kDa_ coating without drug and loaded with 20% PCL_Mw = 2 kDa_, (I) PCL_Mw = 2 kDa:80 kDa_ coating without drug and loaded with 40% PCL_Mw = 2 kDa_, (J) PCL_Mw = 2 kDa:80 kDa_ coating with 10% DXM and loaded with 20% PCL_Mw = 2 kDa_, (K) PCL_Mw = 2 kDa:80 kDa_ coating with 20% DXM and loaded with 20% PCL_Mw = 2 kDa_, (M) PCL_Mw = 2 kDa:60 kDa_ coating with 20% DXM and loaded with 40% PCL_Mw = 2 kDa_). Scale bar = 200 μm (in the above picture); Scale bar = 10 μm (in the following picture).

It was determined that both the thickness and the uniformity of the studied coatings were optimal under the following preparation conditions: one impregnation instance, impregnation time of 10 s, and the PCL concentration of 10%. The morphology of the coatings was assessed macroscopically. All drug-loaded formulations exhibited opalescence, and the presence of DXM rendered the PCL matrix opalescent. However, in the absence of DXM, the coatings were translucent. This fact shows that the drug distribution in the coatings may be relatively uniform. SEM images showed no significant differences between the surface morphologies of unloaded and drug-loaded coatings (Figure 2). These SEM images also confirmed the homogenous distribution of DXM.

### 3.2. Tensile properties of coatings

**Figure 3.**
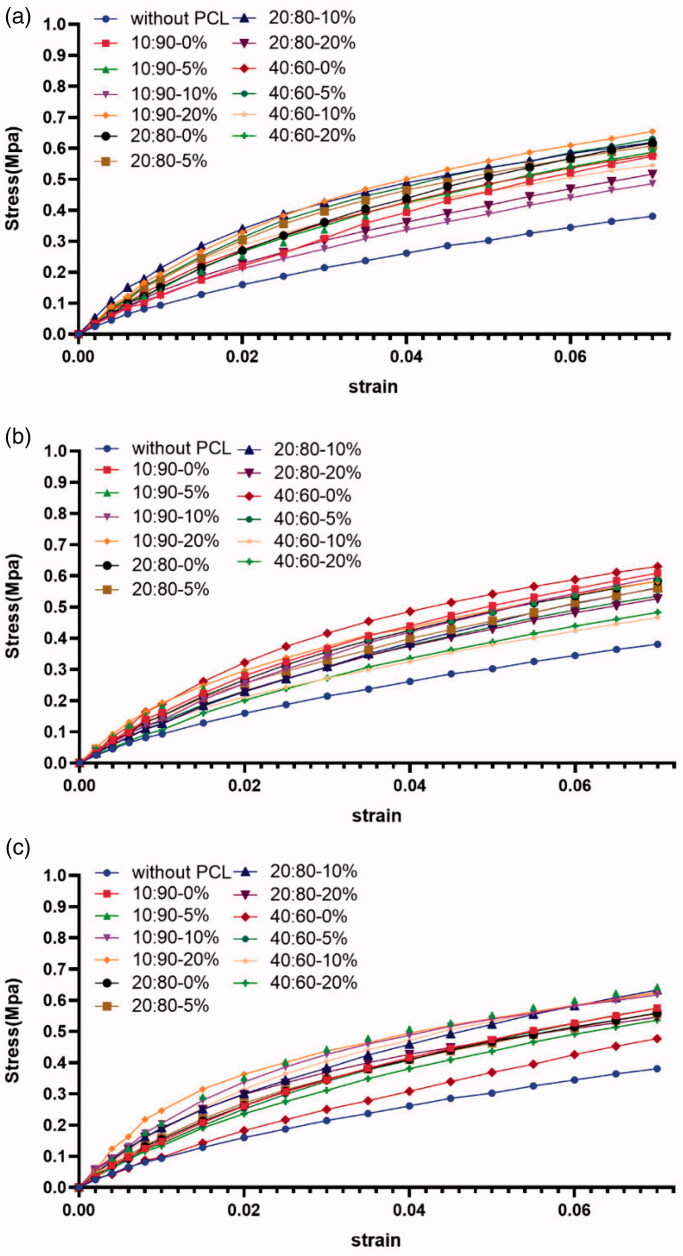
Tensile test profiles, for the different PCL coatings (a. Mw = 2 kDa:36 kDa; b. Mw = 2 kDa:60 kDa; c. Mw = 2 kDa:80 kDa).

The mechanical properties of electrodes are obviously important. A good coating should not affect the flexibility and mechanical stability of its associated electrode. The mechanical properties of the coatings were studied using UTM. The mechanical testing results in the present study are similar to previously reported results. Both PCL and PCL mixture coatings exhibited proper moduli. Importantly, the addition of low-molecular-weight PCL to high-molecular-weight PCL decreased the moduli (Figure 3). The test results showed that the elastic modulus roughly increased with increasing the DXM amount. The force/displacement curve showed similar behavior, irrespective of the formulation ratio. Note that potential alterations after exposure to aqueous media cannot be excluded, and will be addressed in future studies.

### 3.3. *In vitro* DXM release

**Figure 4.**
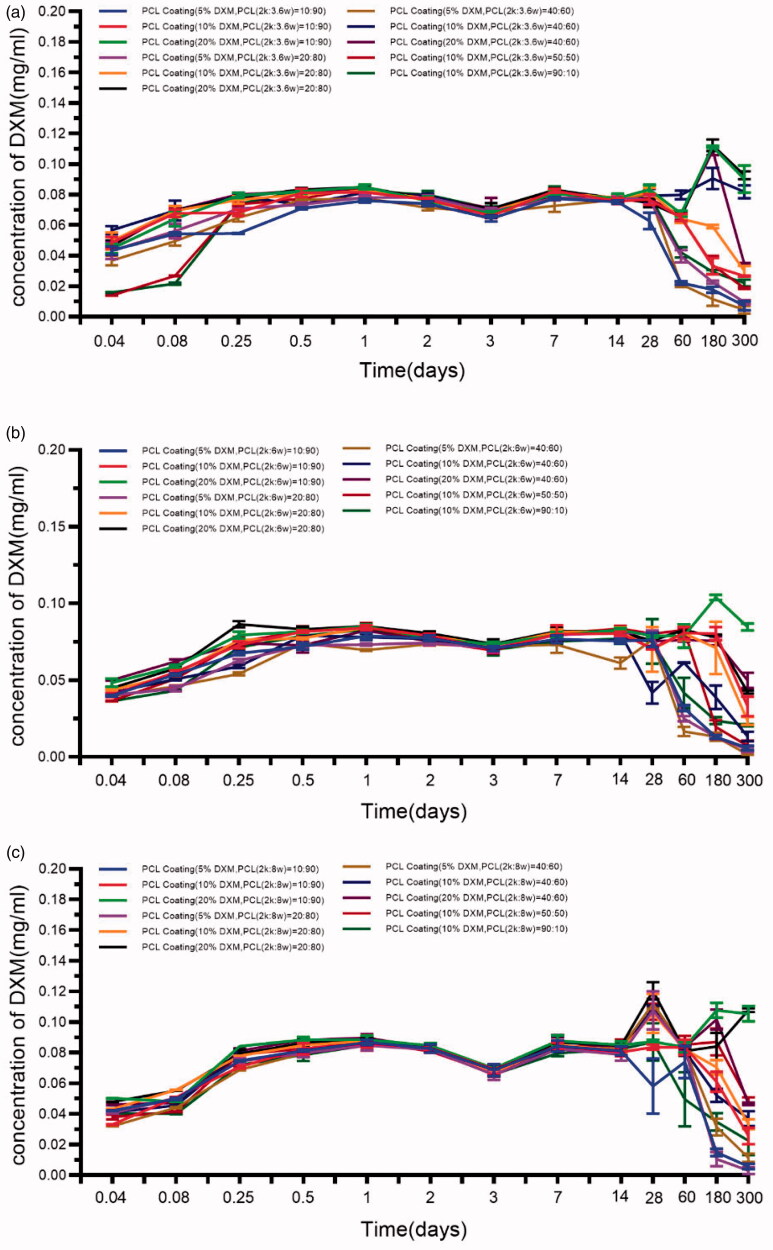
Drug concentration-time profiles, for the different PCL coatings, at pH = 7.4, at 37 °C (a. Mw = 2 kDa:36 kDa; b. Mw = 2 kDa:60 kDa; c. Mw = 2 kDa:80 kDa). The results are expressed as the mean ± S.D. (*n* = 3).

**Figure 5.**
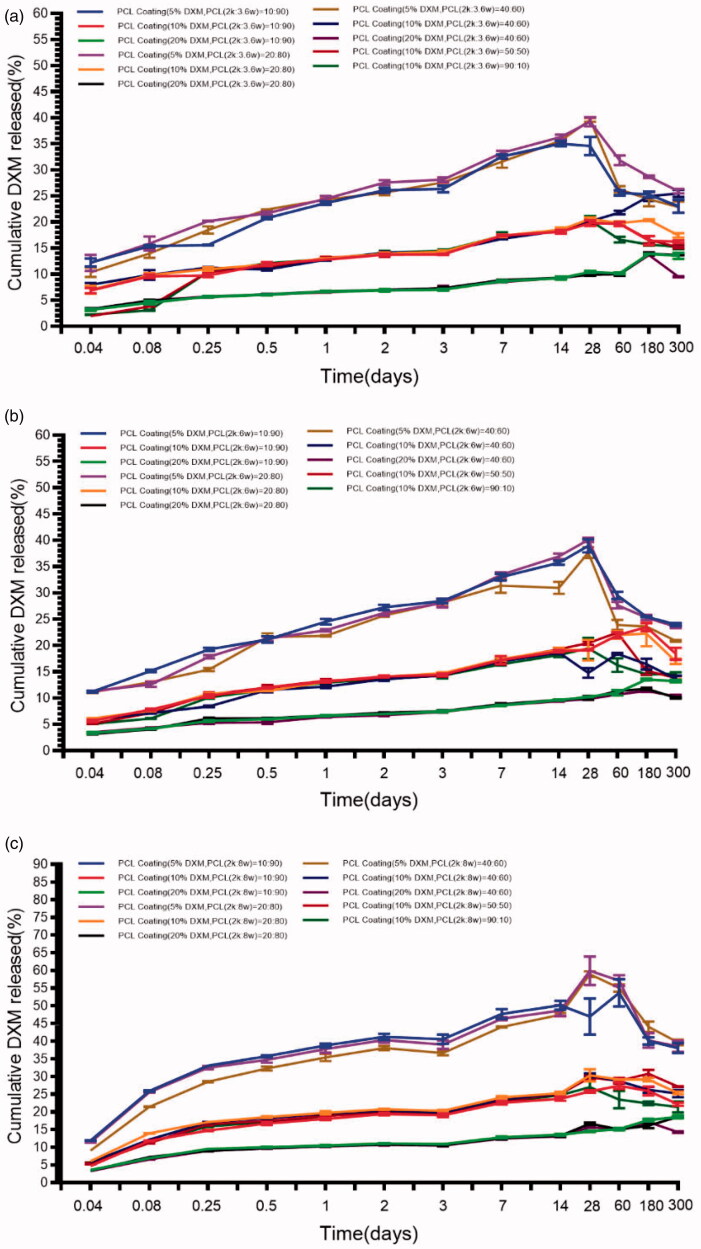
*In vitro* release profiles of DXM from the PCL coatings, at pH = 7.4, at 37 °C (a. Mw = 2 kDa:36 kDa; b. Mw = 2 kDa:60 kDa; c. Mw = 2 kDa:80 kDa). The results are expressed as the mean ± S.D. (*n* = 3).

The release of DXM from the coatings has been investigated at 37 °C in artificial perilymph at pH = 7.4, for 300 days. Figure 4 shows the DXM release profiles/release kinetics. These profiles exhibit biphasic features, with a rapid initial burst-like release followed by a sustained release. Within the first 24 h, the DXM release profiles exhibited the initial burst-like release, which was probably owing to the release of DXM that was entrapped near the coatings’ surfaces. Different amounts of DXM were released from the different coatings within the first 24 h. The following slow release phase might be owing to the drug diffusion. The amount of DXM released from the coatings increased with decreasing DXM (Figure 5). Release profiles with this pattern are quite commonly observed for PCL polymers and have been related to the initial release of DXM adhering to the coating’s surface and the autocatalytic degradation of the PCL. The release profiles were not significantly different from the profiles of single-molecular-weight PCL groups. The high concentration of the initial drug release is in accord with desired kinetic profiles. Compared with the single-molecular-weight PCL, higher initial release rates were observed for all of the analyzed coatings. These results suggest that low-molecular-weight compounds improve the initial release. Importantly, the extent of the initial release can be tuned by tuning the amount of the incorporated low-molecular-weight PCL. Inflammation processes after a typical CI implantation start within a few hours, and the main inflammation phase occurs during the first-week post-implantation (Astolfi et al., 2014). It was shown that drug depots loaded with 20.4 µg DXM protected hearing thresholds and outer hair cell function in implanted animals and lowered the expression of the inflammation marker TNF-a in the cochlear tissue (50 ). A pharmacokinetic study revealed that these silicone-based drug-delivering devices achieved a steady-state drug concentration (∼0.1 µg/mL) one-week post-implantation in the scalar tympani (Liu et al., 2015). The HPLC test results show that all of the formulations can cover this range of concentrations by reaching a steady-state concentration of DXM (range, 16.63–85.2 µg/mL) when using the ratio that covers possible DXM drug loadings of 5%–10%. From the existing literature, we can deduce that the effective dose range of dexamethasone for hearing loss and cochlear injury caused by cochlear implant electrode implantation is from 0.006 mg/ml to 0.016 mg/ml. And the use of dexamethasone treatment should meet both the acute injury after implantation and the long-term damage, so it should be able to release effective drug concentration for up to 1 year. The HPLC results underscore the high potential of these coatings because they can maintain nearly constant drug levels *in vitro* over several weeks, and drug levels can be adjusted to the therapeutic range by dosing the total drug load and the ratio of low-molecular-weight PCL.

### 3.4. *In vitro* safety studies

**Figure 6.**
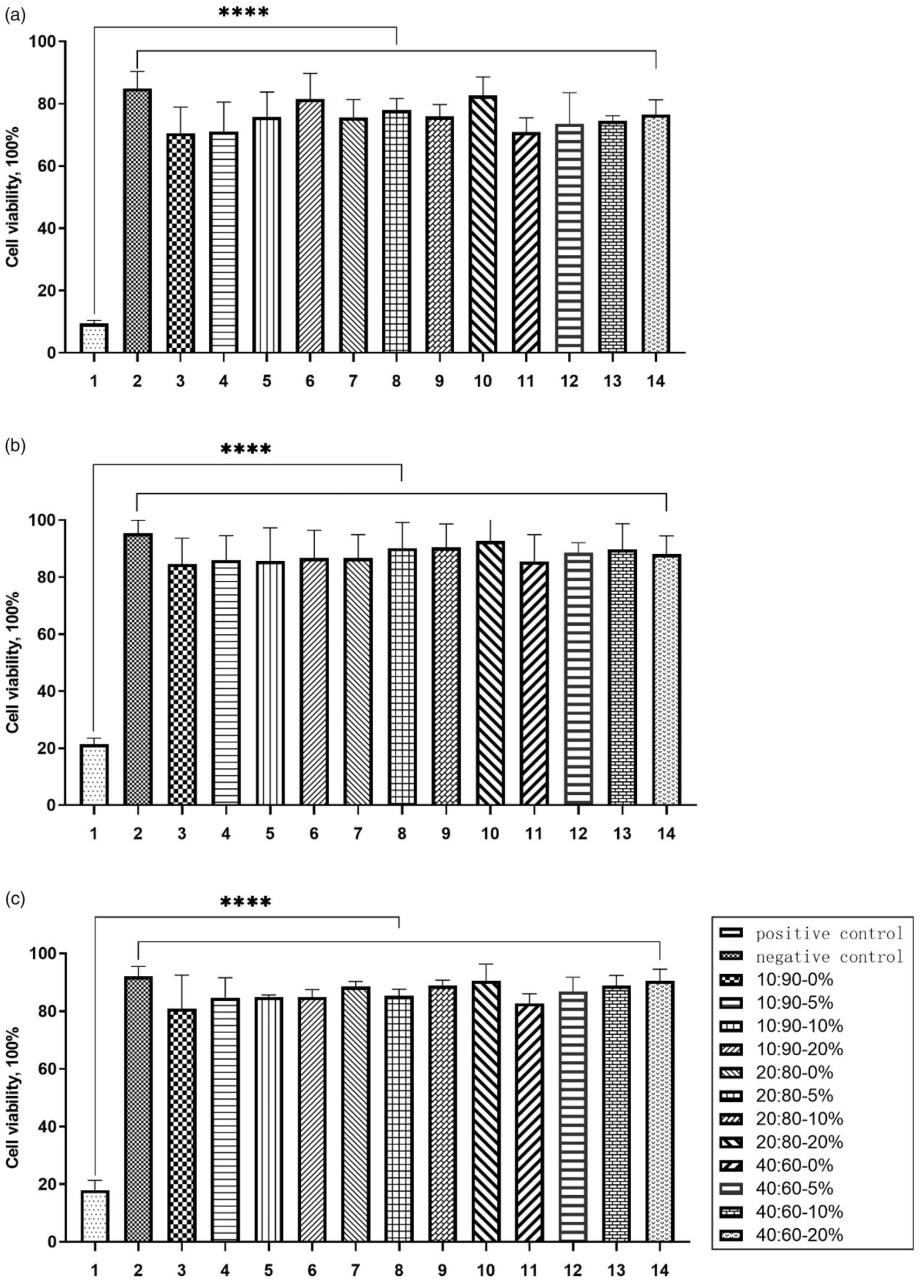
Viability of L929 cells after exposure to the extracted liquid, for the different PCL coatings, at 24 h (a. Mw = 2 kDa:36 kDa; b. Mw = 2 kDa:60 kDa; c. Mw = 2 kDa:80 kDa). The results are expressed as the mean ± S.D. (*n* = 3) (**** *p* < .0001 *vs.* positive control). ****represents that the results of the experimental group and the negative control group are significantly different from those of the positive control group, and p < 0.0001.

To assess the cytotoxicity of the coatings, they were evaluated *in vitro* on L929 mice fibroblasts. The CCK-8 assay was used for measuring the cell viability rate after seeding the cells in the leaching liquor of the coatings for 24 h. L929 cells were cultivated in RPML 1640 in a 96-well plate with filaments. After incubation for 24 h, different leaching liquors of the coatings were cultivated with L929 cells *in vitro* for 24 h; the results are reported below. As shown in Figure 6, the results obtained for all of the leaching liquor groups indicate high cell viability (above 70% for all groups). Importantly, cell viability across the different leaching liquor groups was not significantly different from that of the negative control group (PBS medium) (*p* > .05) but differed significantly from that of the positive control group (cisplatin solution) (*p* < .05). Accordingly, it was concluded that our coatings possess high biological compatibility. These results are in line with previous studies documenting the fine biocompatibility of PCL (Woodruff and Hutmacher, 2010), and confirmed that the preparation process did not lead to any toxicity in the final samples. Importantly, these results demonstrate the great potential of the coatings for future applications.

### 3.5. *In vivo* biocompatibility studies

#### 3.5.1. Gross morphology

The long-time biocompatibility of the coatings with biological tissues was evaluated at 3 weeks post-implantation. During the experiment, all rats remained in good health. For all coatings, no significantly adverse tissue reactions were identified at any time at the implant sites. Importantly, almost all of the coatings showed a complete shape with no breakage.

#### 3.5.2. Histology

**Figure 7.**
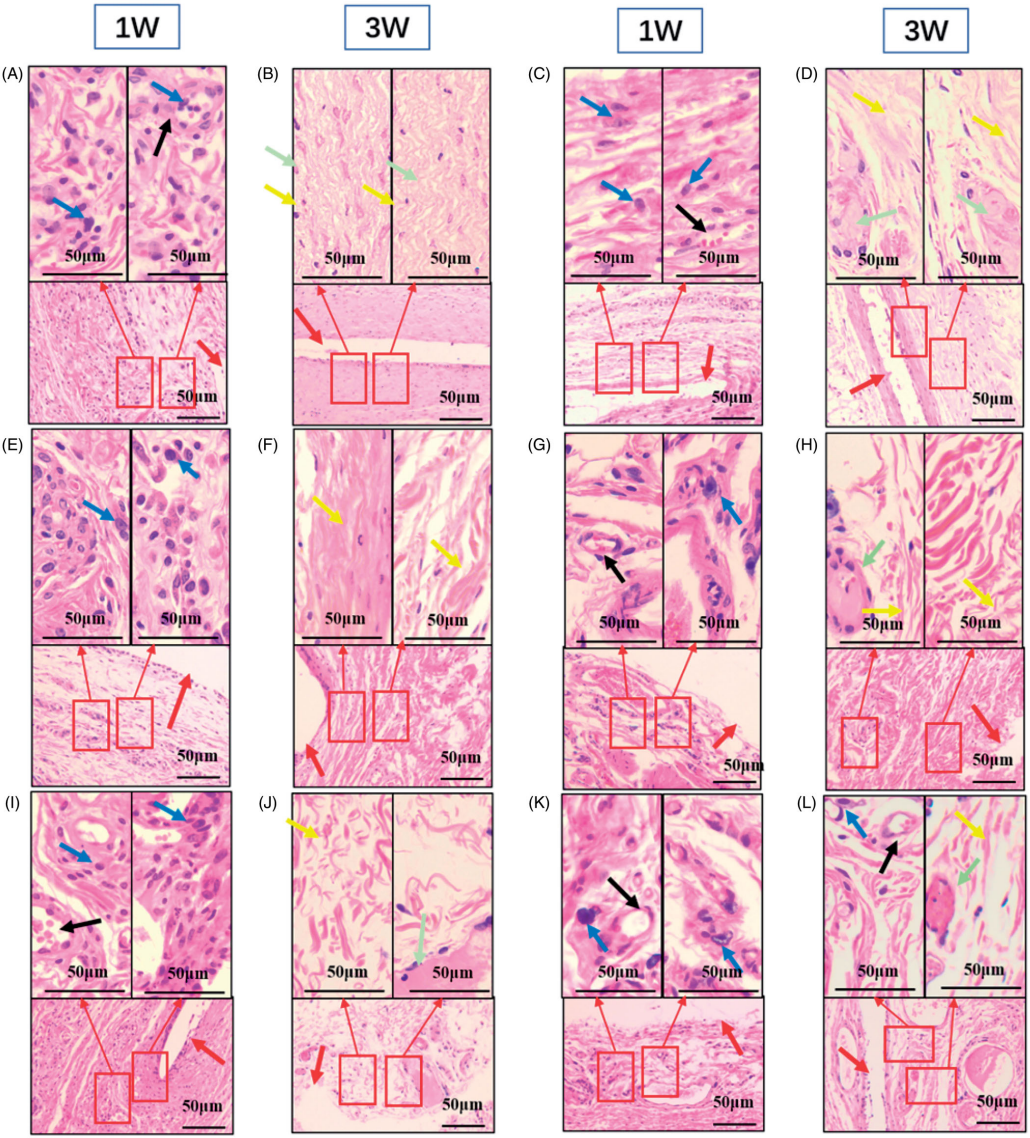
Biocompatibility of the PCL coatings. Hematoxylin and eosin staining suggested the better biocompatibility of the PCL coatings. Scale bar = 50 μm (in the above picture); Scale bar = 50 μm (in the following picture). Neutrophils are designated by blue arrows. Neovascularization is designated by a black arrow. Fibrous tissue is designated by a yellow arrow. Collagen is designated by a green arrow. The implantation site is designated by a red arrow. (A) represents the local tissue one week after the implantation of the coating. The specific composition of the coating is PCL(Mw=2kDa):PCL(Mw=60kDa)=20:80, and there is no drug loaded, (B) represents the local tissue three weeks after the implantation of the coating. The specific composition of the coating is PCL(Mw=2kDa):PCL(Mw=60kDa)=20:80, and there is no drug loaded, (C) represents the local tissue one week after the implantation of the coating. The specific composition of the coating is PCL(Mw=2kDa):PCL(Mw=60kDa)=20:80, and 20% dexamethasone is loaded. (D) represents the local tissue three weeks after the implantation of the coating. The specific composition of the coating is PCL(Mw=2kDa):PCL(Mw=60kDa)=40:60, and 20% dexamethasone is loaded. (E) represents the local tissue one week after the implantation of the coating. The specific composition of the coating is PCL(Mw=2kDa):PCL(Mw=60kDa)=40:60, and 20% dexamethasone is loaded. (F) represents the local tissue three weeks after the implantation of the coating. The specific composition of the coating is PCL(Mw=2kDa):PCL(Mw=60kDa)=40:60, and 20% dexamethasone is loaded. (G) represents the local tissue one week after the implantation of the coating. The specific composition of the coating is PCL(Mw=2kDa):PCL(Mw=60kDa)=40:60, and there is no drug loaded. (H) represents the local tissue three weeks after the implantation of the coating. The specific composition of the coating is PCL(Mw=2kDa):PCL(Mw=60kDa)=40:60, and there is no drug loaded. (I) represents the local tissue one week after the implantation of the coating. The specific composition of the coating is PCL(Mw=2kDa):PCL(Mw=80kDa)=10:90, and there is no drug loaded. (J) represents the local tissue three weeks after the implantation of the coating. The specific composition of the coating is PCL(Mw=2kDa):PCL(Mw=80kDa)=10:90, and there is no drug loaded. (K) represents the local tissue one week after the implantation of the coating. The specific composition of the coating is PCL(Mw=2kDa):PCL(Mw=80kDa)=10:90, and 10% dexamethasone is loaded. (L) represents the local tissue three weeks after the implantation of the coating. The specific composition of the coating is PCL(Mw=2kDa):PCL(Mw=80kDa)=10:90, and 10% dexamethasone is loaded.

**Figure 8.**
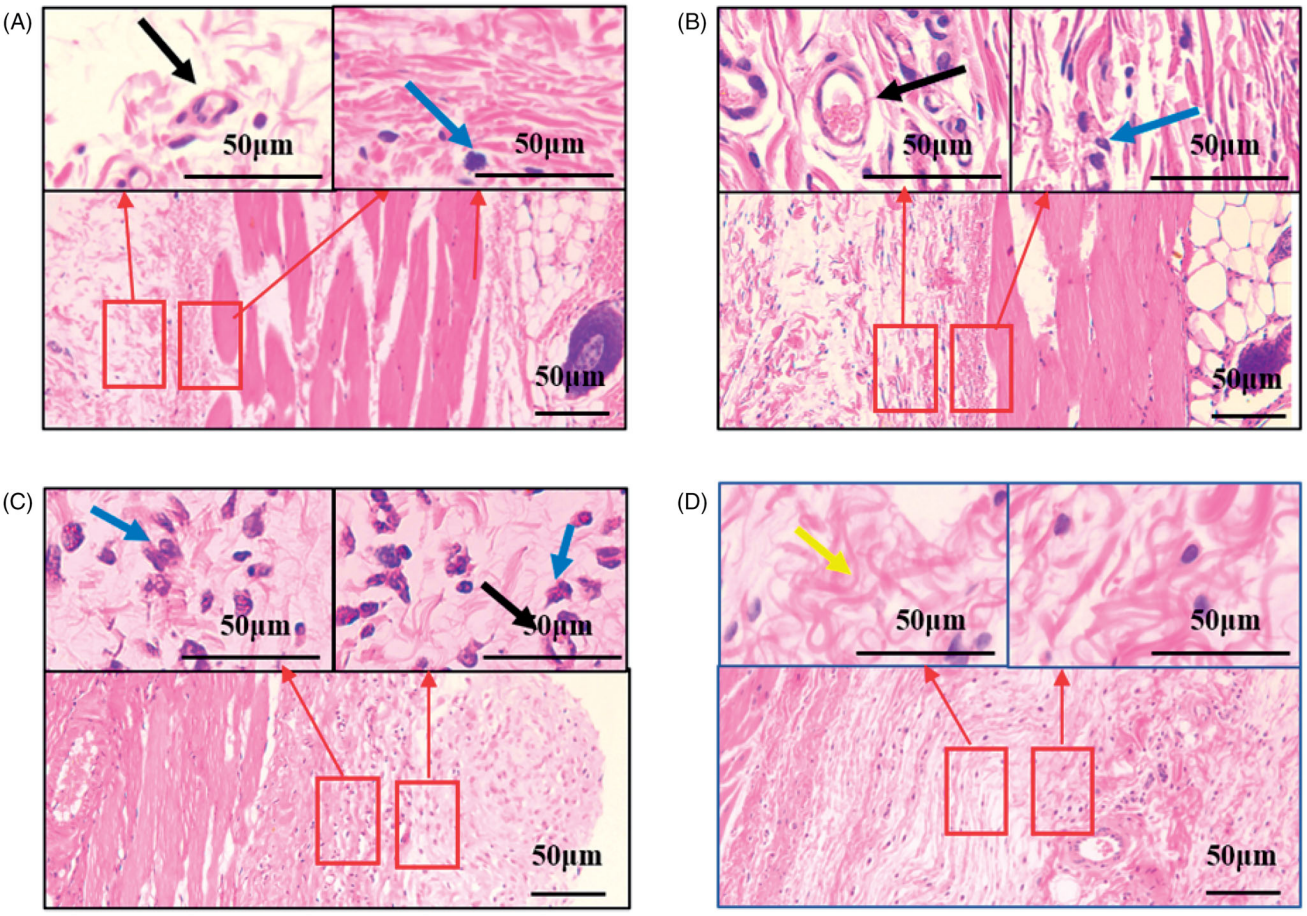
Normal subcutaneous tissue of rat (A,B). Post-operative subcutaneous tissue of rat (C,D). Hematoxylin and eosin staining suggested the better biocompatibility of the PCL coatings. Scale bar = 50 μm (in the above picture); Scale bar = 50 μm (in the following picture). Neutrophils are designated by blue arrows. Neovascularization is designated by a black arrow. Fibrous tissue is designated by a yellow arrow.

**Figure 9.**
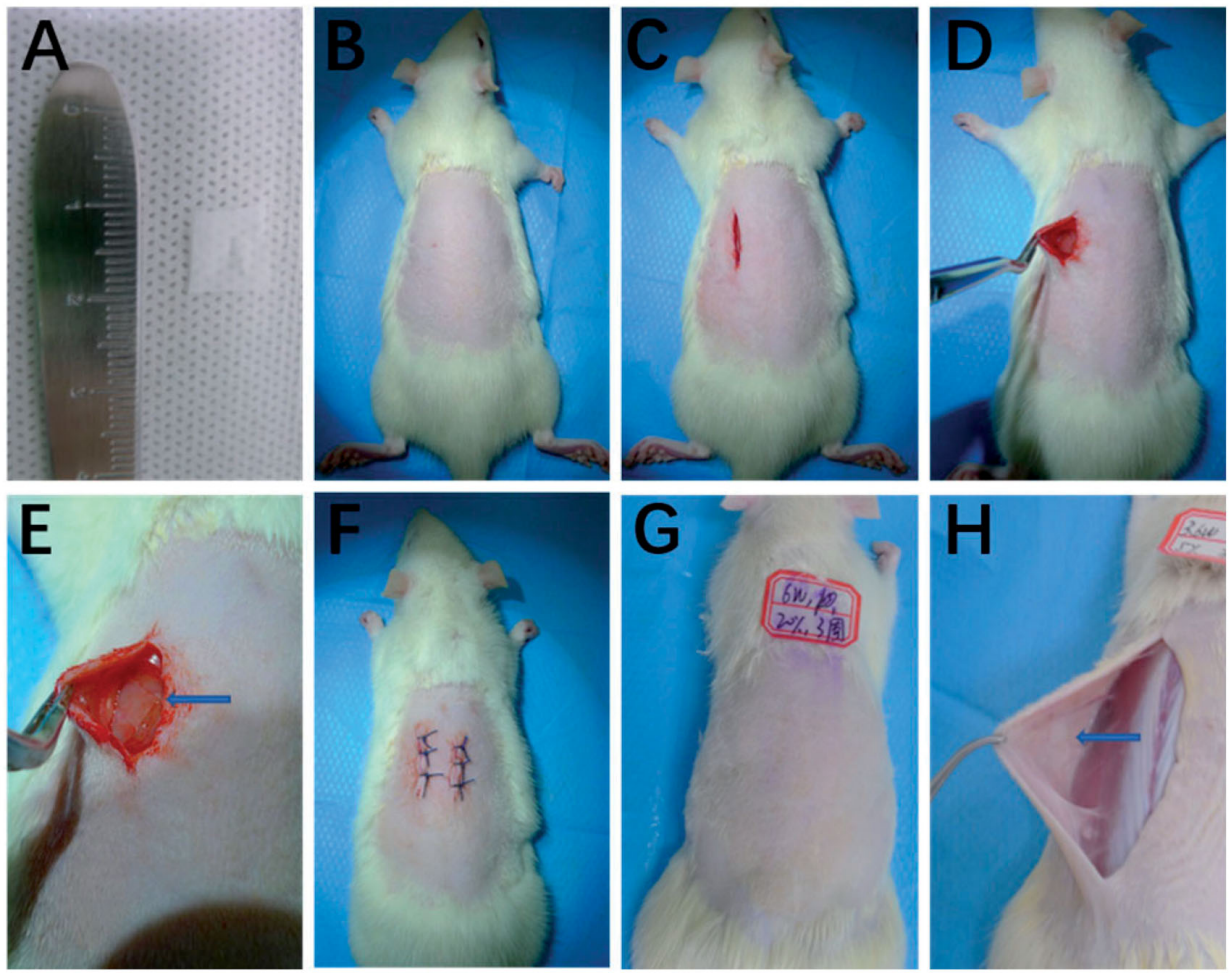
Morphology of the of the PCL coatings (A). Schematic of the implantation operation (B–F). The PCL coatings appear as a white membrane in gross view. The PCL coatings are designated by blue arrows. (G) represents the implantation site of rats three weeks after the implantation of the coating. The specific composition of the coating is PCL(Mw=2kDa):PCL(Mw=60kDa)=10:90, and 20% dexamethasone is loaded, (H) represents the implantation site of rats three weeks after the implantation of the coating. The specific composition of the coating is PCL(Mw=2kDa):PCL(Mw=36kDa)=10:90, and 5% dexamethasone is loaded.

The purpose of the coatings is to reduce the inflammation response to the implant. And ROS is indeed involved in inner ear trauma and inflammation after CI. So, we choose to encapsulate DXM in the preparation of drug-loaded electrode coating. And the mitochondria may play a useful role in biological safety. The high-incidence time period for acute and chronic inflammation is the initial three weeks after the surgery. Host reactions after implantation include injury, acute inflammation, chronic inflammation, foreign body reaction, and fibrosis (Sun et al., 2006). The H&E staining results are shown in Figure 7, at 1-week post-operation. Cell infiltration of the neutrophils at the surgery sites is observed, accompanied by neovascularization. Following acute inflammation, chronic inflammation is identified as a reduction in the number of neutrophils. After 3 weeks post-operation, less neutrophils are observed, an indication of the resolution of the acute inflammatory response to surgery. Importantly, infiltration of neutrophils was weaker for the drug-loaded coatings implant groups than for the surgery groups, at 1 week post-operation (Figure 8). At 3 weeks post-implantation, there were less neutrophils, and more extensive fibrosis was observed based on histological micrographs. Based on the histological micrographs of the coatings at 1 and 3 weeks post-implantation, sustained shape stability was inferred, indicating that the coatings degrade slowly, which is advantageous for reducing the extent of implant-related injury to the cochlea (Figure 9).

## 4. Conclusions

We developed a very thin (thickness, ∼100 μm) PCL-DXM coating composed of various molecular weight PCL blend formulations (ranging from 10% to 40% low-molecular-weight PCL weight ratios). DXM was successfully loaded, for a wide range of values (5–20 wt%). Importantly, the results of *in vitro* release studies suggested that the release of the drug can be controlled, and localized drug delivery is possible. SEM images confirmed the successful fabrication of coatings with uniform and smooth surfaces. From the uniaxial tensile test, we found that the mechanical properties of the coatings depended on the content of the loaded drug. The CCK-8 assay revealed that the coatings had excellent biological compatibility. Subcutaneous implantation in rats showed that the drug-loaded PCL coatings could reduce surgery-induced inflammation. Hence, PCL electrode coatings loaded with 5–20 wt% DXM could be advantageous for localized drug delivery electrode coatings, for preventing and treating inflammation responses caused by mechanical trauma in the implantation process. In addition, these drug delivery coatings may be broadly applicable to any therapeutic approach in which controlled drug delivery is necessary.
